# The Impact of Anaemia on Outcomes, Admissions, and Costs in Patients with Chronic Kidney Disease in Two Public Nephrology Practices in Queensland: A CKD.QLD Registry Study

**DOI:** 10.1155/2023/8720293

**Published:** 2023-05-03

**Authors:** Jianzhen Zhang, Vishal Diwan, Zaimin Wang, Helen G. Healy, Sree Krishna Venuthurupalli, Rajitha Abeysekera, Wendy E. Hoy

**Affiliations:** ^1^NHMRC CKD.CRE and CKD.QLD, Brisbane, QLD, Australia; ^2^Centre for Chronic Disease, The University of Queensland, Brisbane, QLD, Australia; ^3^Kidney Health Services, Metro North Hospital and Health Services, Brisbane, QLD, Australia; ^4^School of Medicine, University of Queensland, Brisbane, QLD, Australia; ^5^Kidney Health Services, West Moreton Hospital and Health Services, Brisbane, QLD, Australia; ^6^Centre for Education Research Training in Kidney Disease, Faculty of Medicine, University of Peradeniya, Peradeniya, Sri Lanka

## Abstract

**Aim:**

Anaemia among patients with chronic kidney disease (CKD) leads to poor overall outcomes. This study explores anaemia and its impact on nondialysis CKD (NDD-CKD) patients.

**Methods:**

2,303 adults with CKD from two CKD.QLD Registry sites were characterised at consent and followed until start of kidney replacement therapy (KRT), death, or censor date. Mean follow-up was 3.9 (SD 2.1) years. Analysis explored the impact of anaemia on death, KRT start, cardiovascular events (CVE), admissions, and costs in these NDD-CKD patients.

**Results:**

At consent, 45.6% patients were anaemic. Males were more often anaemic (53.6%) than females, and anaemia was significantly more common over the age of 65 years. The prevalence of anaemia was highest among CKD patients with diabetic nephropathy (27.4%) and renovascular disease (29.2%) and lowest in patients with genetic renal disease (3.3%). Patients with admissions for gastrointestinal bleeding had more severe anaemia, but accounted for only the minority of cases overall. Administration of ESAs, iron infusions, and blood transfusions were all correlated with more severe degrees of anaemia. The number of hospital admissions, length of stay, and hospital costs were all strikingly higher with more severe degrees of anaemia. Adjusted hazard ratios (CI 95%) of patients with moderate and severe anaemia vs. no anaemia for subsequent CVE, KRT, and death without KRT were 1.7 (1.4–2.0), 2.0 (1.4–2.9), and 1.8 (1.5–2.3), respectively.

**Conclusion:**

Anaemia is associated with higher rates of CVE, progression to KRT and death in NDD- CKD patients, and with greater hospital utilisation and costs. Preventing and treating anaemia should improve clinical and economic outcomes.

## 1. Introduction

Anaemia is common among patients with chronic kidney disease (CKD). Studies indicate approximately 16% of Australians with reduced kidney function have low haemoglobin levels compared to 3.1% among the background population [1]. It is estimated that among the 20 million people with CKD in the United States, approximately 2–4 million (15.4%) have anaemia [2] which is twice the prevalence of the general population (7.6%) [3]. However, the prevalence of anaemia in some large USA CKD cohorts ranges as high as 47% to 78% [4].

Anaemia can be multifactorial with causes such as nutritional deficiencies (iron, B12, folate), relative erythropoietin deficiency, and anaemia of chronic disease being more common. Anaemia results in increased CKD progression rates, cardiovascular events, death, economic burden, and reduced health-related quality of life [5–10].

Management of anaemia in CKD has been through treatment of the underlying cause and the use of erythropoietin-stimulating agents (ESAs). Improved haemoglobin levels with use of ESAs have shown to improve outcomes such as quality of life [11] and requirement for blood transfusions; however, the majority of these data comes from studies conducted among patients on dialysis compared to patients with CKD not on dialysis (nondialysis-dependent chronic kidney disease: NDD-CKD). Importantly, some clinical trials have indicated increased risks for thrombotic events and adverse cardiovascular outcomes with correction of haemoglobin to normal levels, which have raised concerns regarding targets for treatment. Anaemia in CKD tends to be overlooked and goes undertreated in a number of health settings even though effective treatments are clinically available [2, 8], with prescribed treatment reported as low as 30% [3]. CKDopps showed that overall only 48% of patients in the US were treated within 3 months of detecting a Hb < 10 g/dL whilst proportions with any treatment for anaemia in stage 4 and stage 5 NDD-CKD were 24% and 32%, respectively [12].

This study provides more evidence on the characteristics of anaemia in people with NDD CKD; its effect on hospital admissions; and specific outcomes such as cardiovascular events (CVE), conversion to kidney replacement therapy (KRT), all cause deaths, and costs. Results could inform clinical decision-making in NDD-CKD patients.

## 2. Materials and Methods

### 2.1. Study Design and Setting

This is a linkage study on people who had previously given informed consent to participation in the Queensland Chronic Kidney Disease (CKD.QLD) Registry [13]. Specific health clinical and healthcare data were extracted from local and state repositories.

### 2.2. Study Participants

The CKD.QLD Registry is described in detail in a previous publication [14]. In brief, people over the age of 18 years who had been referred to public specialist kidney services in Queensland Health in Australia were deemed by a consultant nephrologist to have established CKD and who were not on KRT were invited to consent to having a defined subset of their clinical data deposited iteratively into the CKD.QLD Registry and be followed over time. Recruitment started in May 2011, and participants were followed until they either reached an endpoint of conversion to KRT or death, or until a censor date of June 2018. This anaemia study includes recruits from two separate geographically and demographically distinct health services run by Queensland Health. These are labelled site A, a metropolitan health service, and site B, a nonmetropolitan health service.

### 2.3. Data Sources

All clinically relevant data were extracted from the CKD.QLD Registry. Linked data on individual patients' health service utilisation and costs between May 2011 and June 2018 were provided by the Queensland Health Statistical Branch from the ^a^Queensland Hospital Admitted Patient Data Collection (QHAPDC) [15], the Queensland Death Registry and the Activity-Based Funding (ABF) Model Output datasets [17].

### 2.4. Data Variables

Anaemia was defined and categorised according to WHO guidelines [18]. The categories included nonanaemia (male ≥ 130 g/L, female ≥ 120 g/L), mild anaemia (male 110−129 g/L, female 110–119), moderate anaemia (80−109 g/L), and severe anaemia (<80 g/L). BMI categories used were underweight (BMI < 18.5 kg/m^2^), normal (BMI 18.5–24.9 kg/m^2^), overweight (BMI 25–29.9 kg/m^2^), mild obesity (BMI 30–34.9 kg/m^2^), and moderate obesity or greater (BMI ≥ 35 kg/m^2^) according to WHO criteria [19]. Urinary protein closest to time of consent was recorded, converted to either albumin to creatinine ratio (ACR) and/or proteinuria to creatinine ratio (PCR) and categorised into normal if ACR <3.4 g/mol or PCR <15 g/mol, microalbuminuria/proteinuria if ACR 3.4–33 g/mol or PCR 15–49 g/mol or macroalbuminuria/proteinuria, and if ACR ≥34 g/mol or PCR ≥50 g/mol [20].

### 2.5. Study Outcomes

The evaluated outcomes were the prevalence of anaemia in the study cohort and its association with hospital admissions, length of stay in hospital, hospital admission costs, cardiovascular events (CVE), gastrointestinal (GI) bleeding, neoplasms, initiation of KRT, and all cause deaths.

CVE were all admissions related to a CVE diagnosis (either as a principal or other reason) and/or admissions related to cardiovascular death. We further classified CVE into three groups: CVE any as identified from both principal diagnosis and/or other diagnosis code sets, CVE principal as identified only from principal diagnosis code set, and CVE associated as identified only from other diagnosis code sets. Death was defined as death from major cardiovascular disease/s according to the International Classification of Diseases, Tenth Revision (ICD–10) I00–I78 [21]. GI bleeding and neoplasms were each similarly classified as any, principal, and associated.

### 2.6. Data Analysis

Stata 16.0 (StataCorp Statistical Software: Release 16.0, College Station. TX 77845: USA, StataCorp LLC, 2019) was used for data analysis. Cox regression was employed to explore the relationship between anaemia severity and primary outcomes, adjusting for potential confounders, which included age, gender, CKD stage, BMI category, proteinuria/albuminuria, site of recruitment, and primary renal diagnosis.

### 2.7. Ethical Approvals

This study was approved by the Royal Brisbane and Women's Hospital Human Research Ethics Committee (HREC/15/QRBW/294) as well as site-specific governance approvals and the University of Queensland Medical Research Ethics Committee (Number: 2011000029). The linked data were accessed through an approved Public Health Act application (RD006802).

## 3. Results

### 3.1. Characteristics of Participants

Table 1 summarises the baseline characteristics of the study cohort. A total of 2,303 CKD patients from the two (2) sites were eligible for the study. There were 1,305 eligible study participants at site A, 52% males, with a mean age of 65.8 years, and with a mean follow-up of 4.2 (SD 2.2) years. There were 998 eligible study participants in site B, 55.6% males, with mean age at consent of 64.2 (SD 15.2) years.

### 3.2. Categories of Anaemia

As shown in Table 1, 54.4% of patients were nonanaemic while 26.7%, 17.8%, and 1% had mild, moderate, and severe anaemia, respectively.  Table 2 summarises the distribution of anaemia by gender, age category, CKD stages, and primary renal diagnosis. The prevalence and severity of anaemia were greater in males than females, and more pronounced at higher ages, especially among those aged >65 years. Anaemia was also more prevalent and severe with more advanced CKD stage. In CKD stages 1–3a, most patients were nonanaemic, while the prevalence and severity of anaemia increased significantly from stages 3b–5. Anaemia was more common and severe among patients with RVD and DN, while the lowest rates were among patients with GRD.

### 3.3. Anaemia and Related Outcomes

**Figure 1:**
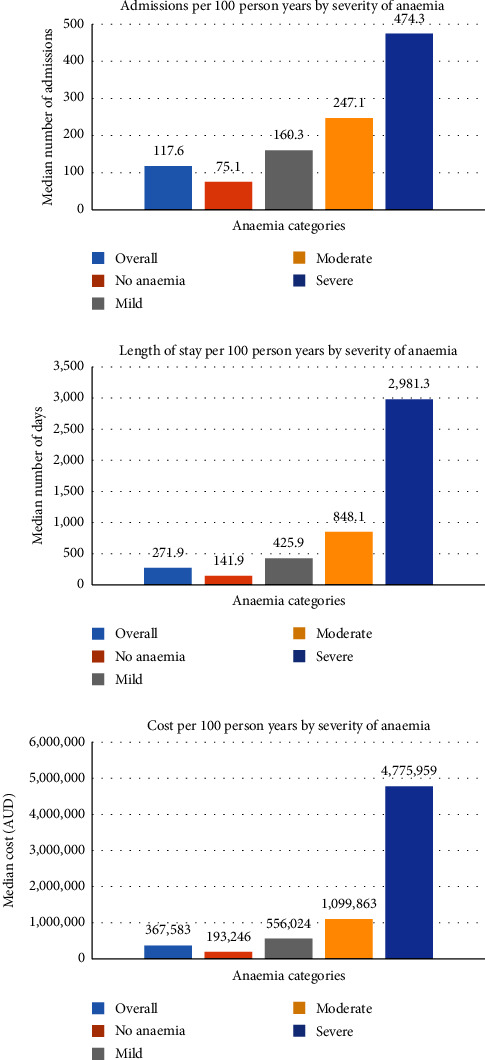
Anaemia and hospital admission related outcomes.

The associations of anaemia with hospital admission numbers, length of stay, and costs among the CKD patients are shown in Figure 1.

#### 3.3.1. Hospital Admissions

A total of 1,972 (85.6%) study participants had at least one hospital admission, with 14,634 total admissions: the median number of hospital admissions was 117.6 (IQR 41.7–280.9) per 100 person years, ranging from 75.1 (25.9–177.9) in the no-anaemia group, and 160.3 (61–337.6), 247.1 (108.6–510.8), and 474.3 (222.4–645.3) in the mild, moderate, and severe categories anaemia groups, respectively. The leading principal cause of admissions was diseases of the circulatory system (*n* = 1,877, 12.8%) (Supplementary data, Table S1).

#### 3.3.2. Hospital Length of Stay

The total length of stay (LOS) was 60,141 days, with a median per person of 9 (IQR 2–31) days. The median hospital length of stay (LOS) per 100 person years was 271.9 (IQR 59.8−1,069.8) days overall, and 141.9 (30.2–605.3) days in those without anaemia, and 425.9 (105.6–1339), 848.1 (241.1–2299.7), and 2,981.6 (1,612.2−7,305) with increasing categories of anaemia.

#### 3.3.3. Hospital Admission Costs

The combined total hospital admission cost of all the participants amounted to AUD 78,900,000. The median cost per participant was AUD 12,401 (IQR 936.00−43,881.22). The total hospital costs per 100 person years were AUD $367,583 (IQR 29,502−1,441,008); it was AUD $193,246 (0–814,278), 556,025 (97,010−1,718,738), 1,099,863 (272,637−3,351,123), and 4,775,959 (1,721,454−9,049,241) in those with nonanaemia, and mild, moderate, and severe degree of anaemia, respectively.

### 3.4. Anaemia Severity and Association with Gastrointestinal Bleeding and Neoplasms

As shown in Table 3, 6.9% of the cohort had admissions for GI bleeding during follow-up: rates were higher with more severe anaemia at baseline, but they still constituted the minority of all anaemic CKD patients. More than quarter of the cohort (27.5%) had admissions for malignancies, but they were not correlated significantly with severity of anaemia at baseline.

### 3.5. Anaemia and Cardiovascular Events (CVE), KRT Starts, and Deaths

Table 4 summarises the patient outcomes of CVE, commencement of KRT, and death without RRT by anaemia severity at baseline.

### 3.6. Cardiovascular Events (CVE)

Of the total participants, 1,067 (46.3%) had CVE as a diagnosis during admission. Among them, 633 (27.5%) had admissions with a principal diagnosis of CVE (with 7,931 admissions), while 973 (42.2%) participants had an associated diagnosis of a CVE (with 6,329 admissions). Compared to patients with no anaemia, all anaemia categories had higher proportion of patients who admitted with CVE as any, principal or associated diagnosis. The incidence rate per 100 person years increased from 8.4 (7.7–9.2) to 15.7 (14.1–17.4) and then to 20.1 (17.7–22.7) among participants who were nonanaemic, mildly anaemic, and moderately/severely anaemic in the overall cohort, respectively. The severity of anaemia was significantly associated (*p* < 0.001) with CVE events after adjusting for the potential confounders, with hazard ratios (95% CI) of 1.4, 1.7 for mild anaemia and moderate/severe anaemia, respectively, compared to those with no anaemia (Table 5).

### 3.7. Commencement of KRT

Among the 2,303 participants, 10.7% (*n* = 247) commenced KRT, with distribution by categories of anaemia summarised in Table 4. The incidence rate of starting KRT per 100 person years increased from 1.2 (0.9–1.5) to 3.9 (3.2–4.9) and then to 7.2 (5.9–8.9) among participants who were nonanaemic, mildly anaemic, and moderately/severely anaemic in the overall cohort. The severity of anaemia was significantly associated (*p* < 0.001) distribution with starting KRT after adjusting for the potential confounders of age, sex, BMI, CKD stage, proteinuria, site of recruitment, and primary diagnosis, with hazard ratios (95% CI) of those with mild anaemia and moderate/severe anaemia compared to those with no anaemia of 1.7 (1.2–2.4) and 2.0 (1.4–2.9) (Table 6).

### 3.8. Death without KRT

Death without KRT occurred in 22.7% (*n* = 523) of the overall study participants. The highest number of deaths were due to diseases of the circulatory system (*n* = 163, 35.5%), neoplasms (*n* = 76, 16.6%), and diabetes-related complications (*n* = 66, 14.4%), respectively, (Supplementary data, Table S2). The proportions of people who died without KRT were 14%, 28.6%, 38.1%, and 65.2% in the no anaemia, mild, moderate, and severe anaemia categories, respectively, (Table 4) and respective incidence rates per 100 person years of 3.2 (2.8–3.7), 7.9 (6.9–9.2), and 13.3 (11.4–15.4). The proportion of people who died without KRT in the moderate/severe anaemia categories was 4 times higher than those without anaemia (13.3 vs. 3.2 per 100 person years), with differences remaining significant after adjusting for age, sex, BMI, CKD stage, primary diagnosis, site of recruitment, and proteinuria (*p* < 0.01). The hazard ratios (95% CI) of those with mild anaemia and moderate/severe anaemia compared to those with no anaemia were 1.3 (1.1–1.7) and 1.8 (1.5–2.3) in the overall cohort (Table 7).

### 3.9. Anaemia-Related Treatment

Table 8 summaries use of treatments of erythropoietin stimulating agents (ESA), iron infusions, and blood transfusions among the study cohort. With worsening degrees of anaemia, the proportion of people who required all three treatment types increased.

## 4. Discussion

Anaemia was common among NDD-CKD patients in two public renal specialty practice sites. Participants with anaemia were significantly older than those without anaemi,a and proportions of patients with anaemia and higher severity of anaemia were significantly higher above the age of 65 years. These findings are comparable to other similar international and national studies [1, 22–27]. Anaemia was more prevalent among males who contrast with the general Australian population: a study in 2011-12 showed that women were more likely to have anaemia than men (6.4% compared with 2.5%) [1]. A higher prevalence of anaemia among males was similarly seen in a US study of people with CKD [28].

The prevalence of anaemia was higher among patients with lower kidney function, which is consistent with the published literature [22, 23], and the highest proportion of patients with anaemia was seen in stages 3b and 4. Underrepresentation of stage 5 CKD patients probably represents the small sample size and earlier survival disadvantage of this group. The CKD cohorts with the highest proportions of anaemia were those with diabetic nephropathy (27.4%) and renovascular disease (29.2%), whereas the lowest prevalence was among patients with genetic renal disease (3.3%). Other studies have reported up to 10 times higher prevalence rates of anaemia among diabetic patients with CKD compared to those without diabetes [7, 22, 29, 30]. While hospital admissions for GI bleeding were more frequent among people with more severe anaemia, only the minority of anaemic persons had such events, and we could find no association of anaemia with neoplasms (Table 3). However, it is worth noting that nearly 27.5% of patients in the study cohort had neoplasm as a comorbidity which highlights the high risk, multimorbid status and more advanced age of many CKD patients, and highlights the complexity and multisystem nature of clinical and holistic care nephrologists and their patients' face.

Evaluation of anaemia and its impact on hospitalisation-related parameters among a CKD cohort not on KRT is a unique aspect in this study and the first of its kind in Australia. Participants with anaemia had higher total number of hospital admissions, longer hospital stay, and higher hospital costs during follow-up than the nonanaemic cohort, and these parameters increased exponentially with worsening degree of anaemia. Nissenson et al. found similar outcomes in a US-insured population with anaemia, with outpatient visits, emergency visits, impatient admissions, LOS, and hospital costs significantly higher than in nonanaemic patients [27]. Smith [6] reported that people with CKD who were anaemic had twice the average annualised costs of CKD without anaemia, while Lefebvre et al. found that nondialysis patients with CKD had an 40% higher overall health care cost than nonanaemic patients, with the largest driver of cost being due to hospitalisations [31]. Further US studies have supported this finding [7].

In our fully adjusted Cox regression models, anaemia was a significant and independent risk factor for CVE, starting KRT, and death. Furthermore, the incident rates and hazards of developing these outcomes significantly increased with worsening anaemia, in keeping the known literature. It is unclear whether anaemia is directly the causative factor or merely associated with adverse outcomes. Many mechanisms such as inflammation, tissue hypoxia, and oxidative stress have been proposed as intermediary mechanisms.

The greater use of ESAs, iron infusions, and blood transfusions in those with more severe anaemia is intuitive; it was not possible to analyse individual responses to these treatments in this study. On a different topic, while the apparently higher rates of CVE outcomes by anaemia categories in site B versus site A participants (hazard ratios 1.4 (*p* < 0.001) are compatible with their higher rates of obesity, diabetes, macroproteinuria, and lower IRSD scores, further evaluation of this phenomenon is needed.

Our findings enrich the knowledge on profiles of anaemia and its influence on the prognosis in NDD-CKD population in Australia. We acknowledge limitations in the study. With the study limited to CKD.QLD Registry patients from two public kidney services in Queensland, the data may not be generalizable to all CKD populations and those from other health environments such as community care. Anaemia was defined from haemoglobin levels at a single time point, and patients' trajectories over the course of the study were not analysed. The number of patients with severe anaemia was few (1%), and a preceding survival disadvantage may have provided underestimates of anaemia-related harms for this category of patients.

In conclusion, anaemia was highly prevalent in these two study CKD cohorts with significant adverse effects on hospitalisations, increased healthcare costs, and poor health outcomes. Mitigating anaemia has the potential to improve clinical and economic outcomes in people with CKD [4]. However, more effective management for anaemia in CKD is needed [32]. This study could provide a baseline for evaluation of better management strategies for NDD-CKD patients [16].

## Figures and Tables

**Table 1:** Baseline characteristics of the two cohorts.

Total participants		Site A	Site B	Overall
		*N* (%)	*N* (%)	*N* (%)
		1,305 (56.7%)	998 (43.3%)	2,303 (100%)
Follow-up (years)	Mean (SD)	4.2 (2.2)	3.4 (1.8)	3.9 (2.1)
	Range	0–7.12	0.01–7.02	0–7.1

Gender	Male	679 (52.0)	555 (55.6)	1234 (53.6)
	Female	626 (47.9)	443 (44.4)	1069 (46.4)

Age (years)	Mean (SD)	65.8 (16.6)	64.2 (15.2)	65.1 (16.0)
	Range	17.7–98.6	18.1–96.3	17.7–98.6

Age categories	<55	308 (23.6)	238 (23.8)	546 (23.7)
	55–<65	183 (14.0)	211 (21.1)	394 (17.1)
	65–<75	370 (28.3)	270 (27.1)	640 (27.8)
	≥75	444 (34.0)	279 (27.9)	723 (31.4)

BMI (kg/m^2^)	Mean (SD)	30.5 (7.9)	31.8 (0.25)	31.1 (8.0)

BMI category	Underweight	15 (1.4)	11 (1.1)	26 (1.3)
	Normal	241 (22.5)	171 (17.1)	412 (19.9)
	Overweight	340 (31.7)	288 (28.9)	628 (30.3)
	Obese	363 (33.9)	398 (39.9)	761 (36.8)
	Morbidly obese	112 (10.4)	130 (13.0)	242 (11.7)

Comorbidities	Diabetes	556 (42.6)	444 (44.5)	1000 (43.4)
	Neoplasms	327 (25.1)	240 (24.0)	567 (24.6)

CKD stage	1	95 (7.3)	68 (6.8)	163 (7.1)
	2	142 (10.9)	126 (12.6)	268 (11.6)
	3a	216 (16.5)	142 (14.2)	358 (15.5)
	3b	417 (31.9)	293 (29.4)	710 (30.8)
	4	361 (27.7)	275 (27.6)	636 (27.6)
	5	74 (5.7)	94 (9.4)	168 (7.3)

Proteinuria/albuminuria category	Normal	370 (29.3)	213 (21.6)	583 (25.9)
	Mild proteinuria	405 (32.1)	268 (27.2)	673 (29.9)
	Macroproteinuria	486 (38.5)	505 (51.2)	991 (44.1)

Haemoglobin (g/L)	Mean (SD)	125.3 (19.7)	127.7(19.7)	126.3 (19.7)

Anaemia category	No anaemia	687 (52.6)	567 (56.8)	1254 (54.4)
	Mild	347 (26.6)	269 (26.9)	616 (26.7)
	Moderate	258 (19.8)	152 (15.2)	410 (17.8)
	Severe	13 (1)	10 (1)	23 (1)

Primary renal diagnosis^*∗*^ (PRD)	RVD^†^	465 (35.6)	174 (17.4)	639 (27.7)
	DN^‡^	232 (17.8)	269 (26.9)	501 (21.7)
	GN^§^	151 (11.6)	154 (15.4)	305 (13.2)
	GRD^¶^	87 (6.7)	39 (3.9)	126 (5.5)
	Other	199 (15.3)	241 (24.1)	440 (19.1)
	Uncertain	171 (13.1)	121 (12.1)	292 (12.7)

Index of Relative Socioeconomic Disadvantage (IRSD)	Lowest	183 (14.1)	297 (29.8)	480 (20.9)
	Low	205 (15.8)	207 (20.7)	412 (17.9)
	Middle	138 (10.6)	417 (41.8)	555 (24.2)
	High	517 (39.8)	29 (2.9)	546 (23.7)
	Highest	255 (19.6)	48 (4.8)	303 ((13.2)

^†^RVD: renovascular disease; ^‡^DN: diabetic nephropathy; ^§^GN: glomerulonephritis; ^¶^GRD: genetic renal disease.

**Table 2:** Anaemia distribution according to baseline characteristics.

	No anaemia	Mild anaemia	Moderate anaemia	Severe anaemia
	*N* (%)	*N* (%)	*N* (%)	*N* (%)
*Gender*				
Male	629 (50.2)	397 (64.4)	195 (47.5)	13 (56.5)
Female	625 (49.8)	219 (35.5)	215 (52.4)	10 (43.4)

*Age category*				
<55 years	392 (31.2)	89 (14.4)	62 (15.1)	3 (13.0)
55–64	227 (18.1)	92 (14.9)	72 (17.5)	3 (13.0)
65–74	340 (27.1)	194 (31.4)	103 (25.2)	3 (13.0)
>75	295 (23.5)	241 (39.1)	173 (42.2)	14 (60.8)

*CKD stage*				
Stage 1	149 (11.8)	10 (1.6)	4 (0.9)	0 (0)
Stage 2	217 (17.3)	43 (6.9)	6 (1.5)	2 (0.7)
Stage 3a	247 (19.7)	82 (13.3)	27 (6.6)	2 (0.7)
Stage 3b	384 (30.6)	205 (33.3)	118 (28.8)	3 (13.0)
Stage 4	230 (18.3)	217 (35.2)	178 (43.4)	11 (47.8)
Stage 5	27 (2.1)	59 (9.6)	77 (18.8)	5 (21.7)

*Primary renal diagnosis (PRD)*				
RVD†	333 (26.6)	183 (29.7)	117 (28.5)	6 (26.1)
DN^‡^	213 (16.9)	152 (24.7)	129 (31.5)	7 (30.4)
GN^§^	187 (14.9)	81 (13.1)	33 (8.0)	4 (17.4)
GRD^¶^	91 (7.3)	23 (3.7)	12 (2.9)	0 (0)
Other	258 (20.6)	109 (17.7)	70 (17.1)	3 (13.0)
Uncertain	172 (13.7)	68 (11.0)	49 (11.9)	3 (13.0)

^†^RVD: renovascular disease; ^‡^DN: diabetic nephropathy; ^§^GN: glomerulonephritis; ^¶^GRD: genetic renal disease.

**Table 3:** Admissions as for GI bleeding and neoplasms by anaemia category at baseline.

	No anaemia *n* (%)	Mild *n* (%)	Moderate *n* (%)	Severe *n* (%)	Total *N* = 2,303	*p* value
*GI bleeding*						
Any diagnosis						
Yes	63 (5.0)	48 (7.8)	41 (10.0)	7 (30.4)	159 (6.9)	<0.01
No	1191 (94.9)	568 (92.2)	369 (90.0)	16 (69.6)	2144 (93.1)	
Principal diagnosis						
Yes	32 (2.6)	26 (4.2)	18 (4.4)	3 (13.0)	79 (3.4)	<0.01
No	1,222 (97.4)	590 (95.8)	392 (95.6)	20 (87.0)	2,224 (96.6)	
Associated diagnosis						
Yes	34 (2.7)	29 (4.7)	31 (7.6)	4 (17.4)	98 (4.3)	<0.01
No	1,220 (97.3)	587 (95.3)	379 (92.4)	19 (82.6)	2,205 (95.7)	

*Neoplasms*						
Any diagnosis						
Yes	343 (27.4)	171 (27.8)	116 (28.3)	4 (17.4)	634 (27.5)	0.72
No	911 (72.6)	445 (72.2)	294 (71.7)	19 (82.6)	1,669 (72.5)	
Primary diagnosis						
Yes	266 (21.2)	133 (21.6)	90 (21.9)	4 (17.4)	493 (21.4)	0.95
No	988 (78.8)	483 (78.4)	320 (78.1)	19 (82.6)	1,810 (78.6)	
Associated diagnosis						
Yes	236 (18.8)	122 (19.8)	98 (23.9)	3 (13.0)	459 (19.9)	0.13
No	1,018 (81.2)	494 (80.2)	312 (76.1)	20 (87.0)	1,844 (80.1)	

**Table 4:** Number of patients who developed primary outcomes.

	No anaemia *N* (%)	Mild anaemia *N* (%)	Moderate anaemia *N* (%)	Severe anaemia *N* (%)	Total *N* (%)
*CVE*					
Any CVE	461 (36.8)	348 (56.5)	245 (59.8)	13 (56.5)	1067/2303 (46.3)
Principal CVE	274 (21.8)	213 (34.6)	140 (34.1)	6 (26.1)	633/2303 (27.5)
Associated CVE	417 (33.2)	315 (51.1)	229 (55.8)	12 (52.2)	973/2303 (42.2)

*Commenced KRT*					
No KRT	1189 (94.8)	527 (85.5)	321 (78.3)	19 (82.6)	2056 (89.3)
KRT start	65 (5.2)	89 (14.4)	89 (21.7)	4 (17.4)	247 (10.7)

*Death without KRT*					
Alive	1078 (85.9)	440 (71.4)	254 (61.9)	8 (34.8)	1780 (77.3)
Death	176 (14)	176 (28.6)	156 (38.1)	15 (65.2)	523 (22.7)

**Table 5:** Cox regression analysis of prediction of CVE by severity of anaemia, adjusting for age, gender, age, BMI, CKD stage, primary renal diagnosis, proteinuria/albuminuria, and site of recruitment.

CV events	Overall HR	*P* value	95%	CI
*Anaemia category*				
Nonanaemia	1.0			
Mild anaemia	1.4	<0.001	1.2	1.6
Moderate & severe anaemia	1.7	<0.001	1.4	2.0

*Age group*				
<55 Years	1.0			
55–64 years	2.3	<0.001	1.7	3.0
65–74	2.5	<0.001	1.9	3.2
≥75	3.4	<0.001	2.6	4.4

*Gender*				
Female	1.0			
Male	1.2	0.01	1.0	1.4

*BMI category*				
Underweight	1.9	0.046	1.0	3.6
Normal	1.0			
Overweight	0.9	0.148	0.7	1.1
Obese	1.0	0.861	0.8	1.2
Morbid obese	1.4	0.004	1.1	1.8

*CKD stage*				
Stage 1&2	1.0			
Stage 3A	1.5	0.010	1.1	1.9
Stage 3B	1.6	<0.001	1.2	2.1
Stage 4	2.3	<0.001	1.7	2.9
Stage 5	4.5	<0.001	3.1	6.4

*Primary renal diagnosis*				
Glomerulonephritis	1.0			
Genetic renal disease	1.3	0.303	0.8	2.3
Renovascular	2.2	<.0.001	1.6	2.9
Diabetic kidney disease	2.6	<0.001	1.9	3.4
Uncertain	1.9	<0.001	1.4	2.7
Others	1.5	0.009	1.1	2.0

*Proteinuria/albuminuria*				
Normal	1.0			
Micro	0.9	0.430	0.8	1.1
Macro	1.4	<0.001	1.2	1.7

*Site*				
Site A	1.0			
Site B	1.4	<0.001	1.2	1.6

**Table 6:** Cox regression analysis of prediction of starting KRT by severity of anaemia, adjusting for age, gender, age, BMI, CKD stage, primary renal diagnosis, proteinuria/albuminuria, and site of recruitment.

Start of KRT	Overall HR	*P* value	95%	CI
*Anaemia category*				
Nonanaemia				
Mild anaemia	1.7	0.004	1.2	2.4
Moderate and severe anaemia	2.0	<0.001	1.4	2.9

*Age group*				
<55 Years	1.0			
55–64 years	0.6	0.002	0.4	0.8
65–74	0.3	<0.001	0.2	0.4
≥75	0.1	<0.001	0.1	0.2

*Gender*				
Female	1.0			
Male	1.7	<0.001	1.3	2.3

*BMI category*				
Underweight	1.1	0.917	0.1	8.2
Normal	1.0			
Overweight	0.9	0.893	0.6	1.4
Obese	1.1	0.377	0.8	1.7
Morbid obese	1.2	0.454	0.7	1.9

*CKD stage*				
Stage 1&2	1.0			
Stage 3A	5.4	0.033	1.1	25.6
Stage 3B	12.9	0.001	3.1	54.6
Stage 4	53.8	<0.001	13.0	222.5
Stage 5	279.3	<0.001	66.3	1176.9

*Primary renal diagnosis*				
Glomerulonephritis	1.0			
Genetic renal disease	2.1	0.014	1.2	3.9
Renovascular	1.0	0.882	0.6	1.8
Diabetic kidney disease	1.3	0.277	0.8	2.0
Uncertain	0.8	0.476	0.4	1.5
Others	1.1	0.793	0.6	1.8

*Proteinuria/albuminuria*				
Normal	1.0			
Micro	1.1	0.718	0.5	2.4
Macro	3.9	<0.001	1.9	7.5

*Site*				
Site A	1.0			
Site B	0.8	0.091	0.6	1.0

**Table 7:** Cox regression analysis of prediction of death without KRT by severity of anaemia, adjusting for age, gender, age, BMI, CKD stage, primary renal diagnosis, proteinuria/albuminuria, and site of recruitment.

Death	Overall HR	*P* value	95%	CI
*Anaemia category*				
Nonanaemia				
Mild anaemia	1.3	<0.013	1.1	1.7
Moderate and severe anaemia	1.8	<0.001	1.5	2.3

*Age group*				
<55 Years	1.0			
55–64 years	2.2	0.004	1.3	3.7
65–74	4.0	<0.001	2.4	6.5
≥75	7.0	<0.001	4.3	11.3

*Gender*				
Female	1.0			
Male	1.2	0.076	0.9	1.4

*BMI category*				
Underweight	1.9	0.067	0.9	4.1
Normal	1.0			
Overweight	0.7	0.013	0.6	0.9
Obese	0.8	0.046	0.6	0.9
Morbid obese	1.2	0.359	0.8	1.7

*CKD stage*				
Stage 1&2	1.0			
Stage 3A	1.2	0.408	0.7	2.1
Stage 3B	1.8	0.014	1.1	2.9
Stage 4	2.9	<0.001	1.8	4.8
Stage 5	5.8	<0.001	3.3	10.3

*Primary renal diagnosis*				
Glomerulonephritis	1.0			
Genetic renal disease	0.8	0.697	0.3	2.3
Renovascular	1.9	0.002	1.3	3.1
Diabetic kidney disease	2.2	<0.001	1.4	3.4
Uncertain	2.2	0.001	1.4	3.6
Others	1.2	0.339	0.8	2.0

*Proteinuria/albuminuria*				
Normal	1.0			
Micro	1.4	0.013	1.1	1.9
Macro	2.0	<0.001	1.5	2.7

*Site*				
Site A	1.0			
Site B	0.9	0.617	0.8	1.2

**Table 8:** Number of patients receiving anaemia-related treatment.

	No anaemia *N* (%)	Mild anaemia *N* (%)	Moderate anaemia *N* (%)	Severe anaemia *N* (%)	Total out of *N* = 2303 *N* (%)
*ESA use*					
Yes	64 (5.1)	133 (21.6)	172 (41.9)	15 (65.2)	384 (16.7)
No	1,190 (94.9)	483 (78.4)	238 (58.1)	8 (34.8)	1,919 (83.3)

*Iron infusions*					
Yes	127 (10.1)	150 (24.3)	156 (38.1)	11 (47.8)	444 (19.3)
No	1,127 (89.9)	466 (75.6)	254 (61.9)	12 (52.2)	1,859 (80.7)

*Blood transfusions*					
Yes	166 (13.2)	182 (29.5)	207 (50.5)	19 (82.6)	574 (24.9)
No	1,088 (86.8)	434 (70.4)	203 (49.5)	4 (17.4)	1,729 (75.1)

## Data Availability

The data used to support the findings of this study are available from the corresponding author upon reasonable request.

## Additional Points

*Summary at a Glance*. This large CKD.QLD Registry-based study identified patterns and rates of anaemia among patients with established chronic kidney disease not on dialysis, and higher rates of progression to kidney replacement therapy, of cardiovascular events and of death as well as greater hospital utilisation and costs in patients with more severe degrees of anaemia.

## Supplementary Materials

Supplementary Materials Supplementary Table S1: principle diagnosis category for hospital admissions (ICD-10-AM). Supplementary Table S2: causes of death by ICD10 diagnosis category.
