# Supplementary material for: The Impact of Anaemia on Outcomes, Admissions, and Costs in Patients with Chronic Kidney Disease in Two Public Nephrology Practices in Queensland: A CKD.QLD Registry Study
Source: Int J Nephrol. 2023 May 3;2023:8720293. doi: 10.1155/2023/8720293 (PMC10171986; doi:10.1155/2023/8720293)
Supplement: Supplementary Materials — Supplementary Table S1: principle diagnosis category for hospital admissions (ICD-10-AM). Supplementary Table S2: causes of death by ICD10 diagnosis category. [file 8720293.f1.pdf]

Supplementary Table S1 – Principle diagnosis category for hospital admissions (ICD-10-AM)

**Diagnosis category**	n (%)
Diseases of the circulatory system	1877 (12.8)
Factors influencing helath status and contact with health services	1617 (11.1)
Symptoms, signs and abnormal clinical and laboratory findings, not elsewhere classified	1223 (8.4)
Neoplasms	1152 (7.9)
Diseases of the genitourinary system	1144 (7.8)
Diseases of the respiratory system	974 (6.7)
Endocrine, nutritional and metabolic diseases	931 (6.4)
Injury, poisoning and certain other consequences of external causes	925 (6.3)
Diseases of the digestive system	918 (6.3)
GI bleeding	*360 (2.5)*
Diseases of the blood and blood-forming organs and certain disorders involving the immune mechanism	822 (5.6)
Diseases of the musculoskeletal system and connective tissue	796 (5.4)
Diseases of the eye and adnexa	761 (5.2)
Diseases of the nervous system	373 (2.5)
Certain infectious and parasitic diseases	341 (2.3)
Diseases of the skin and subcutaneous tissue	309 (2.1)
Mental and behavioral disorders	250 (1.7)
Pregnancy, childbirth and puerperium	117 (0.8)
Diseases of the ear and mastoid process	81 (0.6)
Congenital malformations, deformations and chromosomal abnormalities	19 (0.1)
Certain conditions orginating in the perinatal period	0
External causes of morbidity and mortality	0
Codes for special purposes	0

Supplementary Table S2. Causes of death by ICD10 diagnosis category

	N	%
Diseases of the circulatory system	187	35.8
Neoplasms	89	17.0
Endocrine, nutritional and metabolic diseases	68	13.0
Diseases of the genitourinary system	59	11.3
Diseases of the respiratory system	46	8.8
Diseases of the digestive system	16	3.1
Certain infectious and parasitic diseases	15	2.9
Other	12	2.3
Mental and behavioral disorders	10	1.9
Diseases of the musculoskeletal system and connective tissue	9	1.7
Diseases of the nervous system	7	1.3
Congenital malformations, deformations and chromosomal abnormalities	2	0.4
Diseases of the blood and blood formation organs and certain disorders Involving the immune mechanism	2	0.4
Diseases of the skin and subcutaneous tissue	1	0.2
Total deaths	523	
